# Characterization and Dynamic Shift of Microbial Communities during Start-Up, Overloading and Steady-State in an Anaerobic Membrane Bioreactor

**DOI:** 10.3390/ijerph15071399

**Published:** 2018-07-03

**Authors:** Nsanzumukiza Martin Vincent, Yuansong Wei, Junya Zhang, Dawei Yu, Juan Tong

**Affiliations:** 1 State Key Joint Laboratory of Environmental Simulation and Pollution Control, Research Center for Eco-Environmental Sciences, Chinese Academy of Sciences, Beijing 100085, China; nsanzumumartiv_st@rcees.ac.cn (N.M.V.); zjyzjjzmt@163.com (J.Z.); dwyu@rcees.ac.cn (D.Y.); hittj@163.com (J.T.); 2 Department of Water Pollution Control Technology, Research Center for Eco-Environmental Sciences, Chinese Academy of Sciences, Beijing 100085, China; 3 University of Chinese Academy of Sciences, Beijing 100049, China; 4 Institute of Energy, Jiangxi Academy of Sciences, Nanchang 330096, China

**Keywords:** microbial community shift, bacteria, methanogens, volatile fatty acids (VFAs), anaerobic digestion

## Abstract

A lab-scale anaerobic membrane bioreactor (AnMBR) with a side stream tubular membrane was developed to treat synthetic domestic sewage to evaluate its performance and the dynamic shift of bacterial and archaeal communities during the start-up, steady-state, overloading and recovery periods of operation at mesophilic temperatures. During the start-up period, the bacterial and archaeal communities changed drastically, and *Proteobacteria* and *Bacteroidetes* predominated. During the steady-state period, the AnMBR exhibited excellent COD removal above 91%, and COD of the effluent was below 50 mg/L. High-throughput sequencing analysis results revealed that bacterial and archaeal communities shifted significantly from the start-up to the steady-state period, and that the *Proteobacteria* phylum predominated on days 140, 162 and 190, and the archaea community hydrogenotrophic methanogen genus *Methanolinea* (1.5–6.64%) predominated over the aceticlastic methanogen genus *Methanothrix* (1.35–3.07%). During the overloading period, significant changes occurred in microbial community on day 210, e.g., the phyla *Bacteroidetes* (30%), *Proteobacteria* (23%) and *Firmicutes* (18%) predominated and the archaeal community was completely suppressed, and *Methanobrevibacter* (0.7%) was the only methanogen genus that emerged in the overloading period. After a shock loading period, the microbial communities exhibited significant changes within the ranks of methanogens and shifted to dominance of the aceticlastic methanogen pathway. In addition, the TVFAs to alkalinity ratio in this study was suitable as an indicator of monitoring performance in the AnMBR operation.

## 1. Introduction

Anaerobic digestion systems are considered an attractive option for wastewater treatment because of their manifold advantages [1]; however, conventional anaerobic digestion due to the slow growth rate of anaerobic microorganisms [2] is sensitive to shock loading [3,4] and sludge wash-out caused by short hydraulic retention times (HRTs), sudden temperature drops [4], feeding regime [5,6] and long sludge retention times (SRTs), which reduce the operational flexibility of anaerobic digestion. The start-up of anaerobic digestion is an essential step to establish the proper microbial community structure for anaerobic digestion [7], and an inoculum is necessary in anaerobic digestion [8], to establish a strong microbial community. For preventing sludge washout associated with biomass loss and effectively retain the microbial community in the bioreactor [2], the anaerobic membrane bioreactor (AnMBR) has recently emerged as a potential technology for high-rate anaerobic treatment combining anaerobic biological treatment with membrane filtration, with many advantages including small footprint, decoupling of HRT and SRT, and reduction of sludge production [9]. The AnMBR microbial community structure and diversity are considered to be important factors for the achievement of process steady-state [10]. Apart from this, the AnMBR produces excellent effluent quality in terms of suspended solids, chemical oxygen demand and pathogens, and the produced water is suitable for reuse and recycling for non-potable purposes, compared with the conventional anaerobic processes [11]. Understanding the dynamics and complex interactions of microbial community involved in anaerobic digestion has been a research priority in the last few decades [10,12]. However, the process is driven by complex and dynamic factors, where mechanical, microbiological and physical aspects can all influence the performance [13]. The occurrence of frequent shock loading affects the microbial community structure and provokes microbial significant shift, and strongly affects the methanogen populations, as methanogens are relatively sensitive to sudden environmental changes [6]. Disruption of the methanogen community provokes the sudden accumulation of volatile fatty acids (VFAs) in anaerobic digestion processes. Previous studies revealed that conventional anaerobic digestion systems with a high population of aceticlastic methanogens (*Methanosarcina* and *Methanosaeta*) recover more quickly from shock than anaerobic systems with hydrogenotrophic populations [14,15]. However, there is a missing gap of effects of start-up and shock loading on microbial community of the AnMBR.

Therefore the purposes of this study were to investigate the characterization and dynamic shift of complex anaerobic microbial community including the archaeal and bacterial community structures during the start-up, stable operation, overloading and recovery periods in an AnMBR treating synthetic domestic wastewater at mesophilic temperature, through using a high-throughput sequencing method. Multivariate statistical analysis was also applied for further understanding the relationship of microbial communities over time.

## 2. Materials and Methods

### 2.1. Anaerobic Membrane Bioreactor Operation

**Figure 1:**
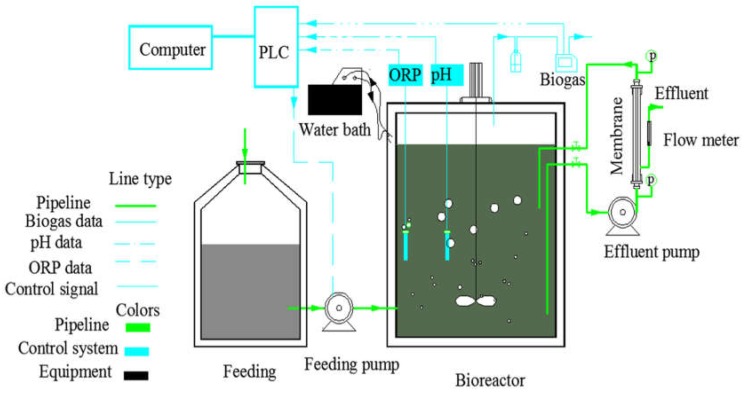
Diagram of the lab scale side-stream anaerobic membrane bioreactor (AnMBR). ORP, oxidation reduction potential; p: pressure gauge; PLC, programmable logic controller.

A side-stream AnMBR with a bioreactor with working volume of 29 L was used to treat synthetic domestic wastewater (Figure 1). Solid-liquid separation was achieved using a tubular microfiltration membrane module with a total surface area of 0.011 m^2^ (PVDF, pore size 0.1 µm, tubular membrane, Beijing Tri-High Membrane Technology Co., Ltd., Beijing, China) through a pump (Peristaltic pumps, BT100-2J, Longer Precision Pump Co., Ltd., Hebei, China) controlled by a programmable logic controller (PLC) system (SIMATIC S7-200CPU224XP, Siemens AG, Munich, Germany). Bioreactor operation and data acquisition were controlled by the PLC system. The bioreactor temperature was kept in the mesophilic range (36–37.5 °C) with a water bath system (Water bath, Ningbo Scientz Biotechnology Co., Ltd., Ningbo, China) connected to the bioreactor. The pH was in the range of 7.2–8.5 during the treatment process. The control program was responsible for operation of all pumps, mixing and online sensors, including the pH sensor (E201-C, Leici Instrument Incorporated, Shanghai, China) and oxidation reduction potential (ORP) probe (HBM-100A, DKK-TOA Corporation, Tokyo, Japan).

### 2.2. Inoculum, Sample Collection and Operational Parameters of the AnMBR

The AnMBR was inoculated with seed sludge at 20 g/L of MLSS from a brewery wastewater treatment plant in the start-up period, and after shock loading, a new inoculum with 16.14 g/L of MLSS from an anaerobic plant in the Xiao Hong Men municipal wastewater treatment plant was used to prevent the AnMBR from failing. During the operational period, the AnMBR was fed with synthetic wastewater with a chemical oxygen demand (COD) concentration in the range of 450–650 mg/L.

The ammonium concentration in the influent was between 45 and 50 mg/L. Synthetic domestic wastewater was prepared using glucose and sodium acetate as the carbon sources and ammonium chloride as the ammonia source. Sodium hydrogen carbonate was used to buffer the system, and the synthetic domestic wastewater contained some trace elements for the growth of bacteria as reported in [16]. The initial volumetric organic loading rate (OLR) was set at 0.25 kg COD/m^3^/day (OLR), which corresponded to a hydraulic retention time (HRT) of 37.5 h. For microbial community analysis, samples of 40 mL of sludge from the AnMBR were taken from the recirculation loop to obtain representative samples, which were stored at −80 °C for further analysis. Twelve samples were collected from the start-up, steady-state, overloading and recovery periods of the AnMBR process. During the start-up period, two samples were collected on days 70 and 100, respectively. During the steady-state period of the bioreactor, three samples were collected on days 140, 162 and 190, respectively. When the AnMBR was in the overloading phase, one sample was collected on day 210, and twenty days later a second sample was collected to further understand this phenomenon, as a new inoculum was used to prevent the AnMBR process from failing. During the recovery process, on days 245, 250, 260, 275 and 285, respectively, five samples were collected.

Twelve samples collected in the bioreactor from the start-up, steady-state, overloading and recovery periods of the AnMBR process were analyzed. The seed sample (Seed 1) was used at the start-up on day zero (2 September 2015), and two samples (A2 and B3) were collected on days 70 (10 November 2015) and day 100 (20 December 2015) during the start-up period, respectively. At the steady-state period, three samples (C4, D5 and E8) were collected on days 140 (13 January 2016), 162 (23 March 2016) and 190 (20 April 2016), respectively. When the AnMBR faced hydraulic overloading due to the technical failure, one sample (F10) was collected on day 210 (29 April 2016), and after twenty days later on day 230 (21 May 2016), a second sample (J15) was collected to further understand this phenomenon, as a new inoculum (Seed 2) was used on the day 220 (10 May 2016) to prevent the AnMBR process from failing. During the recovery process, on days 245 (8 June 2016), 250 (15 June 2016), 260 (25 June 2016), 275 (10 July 2016) and 285 (25 July 2016), five samples (K16, L18, M20, N23 and M24) were collected, respectively.

### 2.3. Microbial Community Analysis

DNA was extracted using a soil total DNA kit (MP Biomedicals, Solon, OH, USA) in accordance with the manufacturer’s instructions. Samples from the bioreactor start-up period to the steady-state period and disturbance to the recovery period were chosen for bacterial and archaeal community analysis. Sludge samples of 1 mL each were analyzed with a FASTDNA Spin Kit for Soil (MP Biomedicals) in duplicate according to the manufacturer’s instructions. The quality and concentration of the extracted DNA were determined using 1% agarose gel electrophoresis and a Nano Drop ND-1000 spectrometer (BioSuplus, San Diego, CA, USA), respectively. PCR primers 515F and 806R targeting both bacteria and archaea 16S rRNA V4 regions were selected for bacterial and archaeal community analysis [17]. The reverse primer contained a unique 6-bp error-correcting barcode for every sample. The barcode was permuted for each sample, which allowed the determination of individual samples within a mixture in a single Illumina MiSeq sequencing run. DNA was amplified in triplicate for every sample. PCR amplicons were then purified; the concentrations were measured by spectrometry using a QuantiFluor^TM^-ST instrument (Promega, Madison, WI, USA). Amplicons from all samples were mixed to obtain equal mass concentrations in the final results, then were sent out to Sangon Co., Ltd. (Shanghai, China) for small-fragment library construction and pair-end sequencing using the Illumina MiSeq sequencing system (Illumina, Albany, NY, USA). Sequencing reads were related to each other among the 14 samples according to the unique 6-bp barcode for every sample. Pairs of reads corresponding to the original copy of DNA fragments were merged using FLASH and filtered using QIIME quality filters. PCR chimeras were filtered using UCHIME, and the remaining sequences were normalized to correctly balance the samples of the same sequencing depth. The normalized sequences have been submitted to the NCBI Sequence Read Archive (SRA) under the project number PRJNA433291. The taxonomic classification of the sequences of 14 AnMBR samples was carried out using RDP classifier.

### 2.4. Analytical Methods

VFAs (acetic, propionic, *i*-butyric, *n*-butyric, *i*-valeric and *n*-valeric) were analyzed by a gas chromatograph (GC, 4890D, Agilent Inc., Santa Clara, CA, USA) equipped with a Flame Ionization Detector (HP, 7673A, Hewlett-Packard, Palo Alto, CA, USA). COD and NH_4_^+^-N were determined according to standard methods [18]. The anions elements such as SO_4_^2−^ were measured with an Ion Chromatography (ICS-1000, Dionex, Sunnyvale, CA, USA). In order to analyze the similarity of the AnMBR microbial community under different operational conditions, principle component analysis (PCA) was conducted in this study, and redundancy analysis was carried out using the software package Canoco version 5 (http://www.canoco5.com). The biogas was collected from bioreactor through a pipe and measured by a biogas flow meter (µFlow, Bioprocess Control AB, Stockholm, Sweden). The ammonia gas (NH_3_) and hydrogen sulfide (H_2_S) were assessed using an Ammonia 2/a 6733231 Draeger Tube and Hydrogen Sulfide 0.2/a 8101461 Draeger Tube (Draeger Safety, Inc., Houston, TX, USA), respectively.

## 3. Results

### 3.1. Performance of the AnMBR Bioreactor

**Figure 2:**
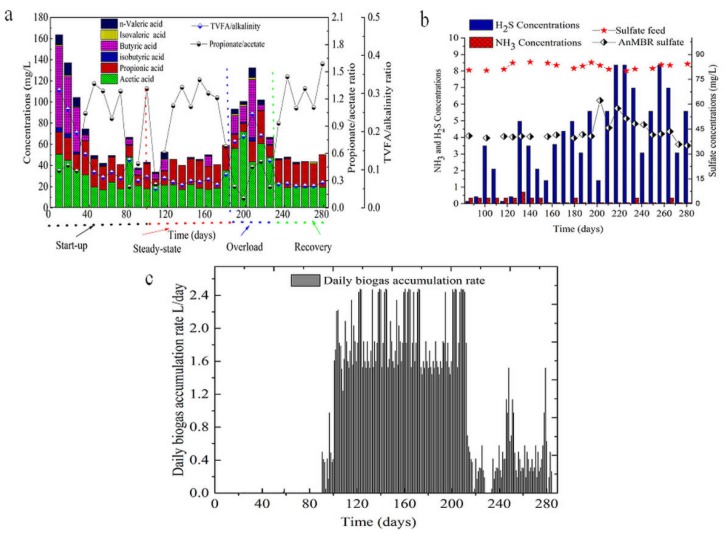
Total volatile fatty acids (TVFA) (**a**), Composition of NH_3_, H_2_S and sulfate concentrations (**b**), Daily cumulative biogas production (**c**) in AnMBR over time fluctuation during AnMBR process (**b**), RE: Removal efficiency; sCOD: soluble chemical oxygen demand.

The AnMBR operated for approximately 285 days, at HRT of 37 h and SRT of 100 days, respectively. The AnMBR performance was evaluated in terms of organic matter removal efficiency (i.e., COD) and VFAs reduction. During the start-up period, the organic matter removal efficiency was very low, and the concentration of TVFAs in the AnMBR was in the range of 104–163 mg/L, and the pH and ORP fluctuated in range of 7.72–7.90 and −160 to −175 mv, respectively. During the steady-state period, the average soluble chemical oxygen demand (sCOD) removal efficiency reached above 91% ± 6.8%, with an average effluent sCOD concentration of 45 ± 4.8 mg/L. In this period, a low concentration of VFAs was recorded in the AnMBR as shown in Figure 2a, and the TVFAs to alkalinity ratio was 0.11. In general, the amount of TVFAs in the AnMBR at steady-state was below 60 mg/L, which was favorable for the good performance (Figure 2a). The AnMBR alkalinity was 675.25 ± 188.42 mg/L CaCO_3_, sufficient for sustaining the anaerobic microbial community. Around day 210, the AnMBR faced hydraulic overloading due to the technical failure of the logic program (Figure S1), and its performance dropped dramatically while the concentration of TVFAs increased. During the shock loading on day 210, the total TVFAs accumulation in the AnMBR reached above 132 mg/L.

In this period the average alkalinity concentration was 537.33 ± 238.28 mg/L CaCO_3_, the propionate to acetate ratio decreased drastically in the range of 0.10–0.50. In this study, in the start-up and shock loading stages, a high concentration of butyric acid occurred and only lasted for a few days in the AnMBR (Figure 2a), but a high concentration of acetate persisted throughout the periods. This phenomenon induced the methanogens and bacteria to shift and caused a change in the microbial community structure. The pH value was maintained in the range of 6.9–7.9. Inanc et al. (1999) indicated that propionic producer benefited around neutral pH and high substrate level [19], similar to the findings of this study on day 210 and 230 (Table 1), where after shock loading, a pH of 7.20 near neutrality was observed. During the AnMBR recovery, the TVFAs concentration decreased gradually towards below 60 mg/L and the alkalinity in the bioreactor was in the range of 758.00 ± 136.53 (mg/L) CaCO_3_. As shown in Figure 2c the daily cumulative biogas production of this AnMBR was above 1.6 L/day in the steady-state period, than in the recovery period, above 0.5 L/day. As listed in Table 1, the pH and ORP for different stages were stable for the start-up, steady-state and recovery operation, a slight change of ORP and pH only occurred during the hydraulic shock loading. The daily biogas production was insignificant during the first days of the AnMBR operation, mainly due to the low activity of the methanogen community. After the start-up period of 100 days, the daily biogas production rate increased to 1.85 ± 0.45 L/day, and hydrogen sulfide (H_2_S) gas was also recorded at low concentrations, except on days between 160 and 180 mg/L (Figure 2b). At the hydraulic shock, the biogas production was suppressed as shown in Figure 2c. As shown in Figure 2b, the sulfate (SO_4_^2−^) concentration in feed was below 85 mg/L, and results of the H_2_S in the biogas showed the reduction of sulfate and increases of sulfate reducing bacteria in the AnMBR.

After the overloading period, the daily biogas production decreased considerably to below 0.48 ± 0.33 L/day, probably due to inhibition of methanogen community after overloading. Besides, the decrease in the daily biogas production found during operation of the AnMBR can be associated with the presence of a high relative abundance of sulfate-reducing bacteria, which can compete with methanogens for substrate consumption [20]. In the steady-state period, *Desulfovibrio* presented low relative abundance between 0.04% and 0.4%; however, after the overloading period, this genus increased to a relative abundance between 0.06 and 1.27%, and such high relative abundance of sulfate-reducing bacteria was correlated to the high amount of hydrogen sulfide gas recorded (Figure 2c) and low daily biogas accumulation in the AnMBR (Figure 2c).

### 3.2. Microbial Community Evolution

#### 3.2.1. Microbial Community Composition and Shift

**Figure 3:**
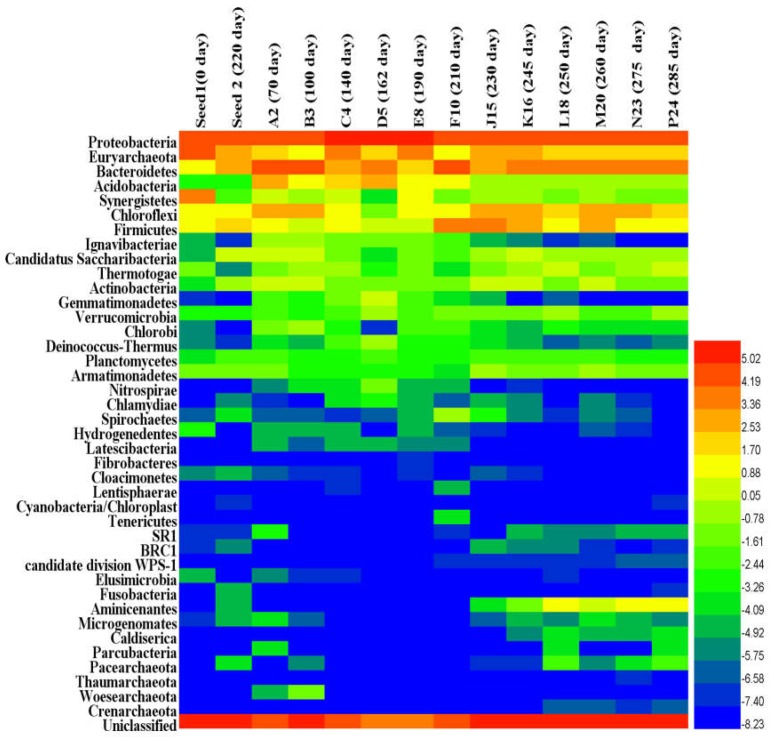
Heat map of the relative abundances of various phyla (>0.01%) across all 12 AnMBR and two inoculum samples (the values were log 2 transformed).

The phyla profile patterns of bacteria and archaea are shown in Figure 3. A clear shift in the prokaryotic community was observed in the AnMBR from the start-up to the steady-state, overloading and recovery operation phases. The inoculum composition was dominated with *Euryarchaeota* (28.08%), *Proteobacteria* (18.84%), and *Synerigestetes* (10.67%) phyla. At the start-up period, microbial community exhibited a significant shift in leading phyla, and the dominant phyla on day 70 were *Bacteroidetes*, *Proteobacteria*, *Chloroflexi* and *Acidobacteria*, but the emergence of some phyla affiliated with the *Nitrospirae*, *Candidatus*, *Chlamydiae* and *Chlorobia* were newly detected in the AnMBR (Table S1), which emphasized the significant shift and adaptation of the microbial community. On day 100, microbial community exhibited continuous shift of the leading phyla, which were mainly represented with Unclassified, *Proteobacteria*, *Bacteroidetes* and *Chloroflexi*. *Bacteroidetes* decreased along with the time, which can be considered as an early sign of process towards the steady-state. After about 100 days of operation, AnMBR achieved the steady-state, which was reflected by the high COD removal (>91%) and low TVFAs levels (<60 mg/L) in bioreactor.

The samples collected on days 140, 162 and 190, phyla were mainly represented by *Proteobacteria* (45–65%), unclassified (10–25%), *Euryarchaeota* (4–12%) and *Bacteroidetes* (5–13%), however, on the day 162, *Bacteroidetes* and *Acidobacteria* increased sharply from 5.6% to 2.7% on day 140 to 13% and 8%, respectively. The phyla of *Actinobacteria*, *Candidatus* and *Chlorobi* were represented with low relative abundance, especially *Chloroflexi* and *Synergistetes* on day 165 while several unforeseen species emerged such as *Deinococcus-Thermus*, *Verrucomicrobia* and *Gemmatimonadetes* (Table S1).

On day 210, the AnMBR experienced a shock loading due to the malfunction of the logic program that led to the entire microbial community structure changing, and a significant shift of the phyla was observed (Figure 3). The AnMBR organic matter removal performance dropped drastically to about 76.50%, and the fermentative *Bacteroidete* (30.05%) and *Firmicutes* (16.00%) predominated. The TVFAs concentration in bioreactor was above 120 mg/L, the acetic and butyric acids increased significantly (Figure 2a). The *Bacteroidetes* and *Firmicutes* outcompeted others phyla. Unforeseen phyla such as *Spirochete* appeared during the overloading process (Table S1) and disappeared again after the VFAs concentration was stabilized in the AnMBR. At this stage, *Proteobacteria* (25.06%), *Firmicutes* (13.30%), *Bacteroidetes* (8.02%), *Chloroflexi* (7.70%) and *Euryarchaeota* (6.42%) were the most dominant phyla (Figure 3). Some phyla exhibited an increase during this transition period such as *Chloroflexi* and *Euryarchaeota* (Table S1). The *Proteobacteria* population remained dynamically stable from day 210 on, while the *Bacteroidetes* and *Firmicutes* population decreased from the day 230 to 245 as the AnMBR was heading to the recovery state. On the days 250, 260, 275 and 285, five phyla—*Proteobacteria* (23–28%), *Bacteroidetes* (10–12%), *Chloroflexi* (4–7%) and *Euryarchaeota* (4–5%)—predominated. Several new species emerged at this period, such as *Aminicenantes* (1.2–2.5%) and *Thermotogae* (0.7–0.9%) (Table S1), which illustrated a partial recovery to some degree (Figure 3).

#### 3.2.2. Archaea and Bacteria Dynamic Populations at the Genus and Family

Archaea and bacteria were characterized at the genus and family levels from the start-up, through the steady-state, shock loading and recovery periods. The archaeal levels were low in all steps since this AnMBR exhibited low biogas production due to low COD in the feed. In the start-up period, methanogen abundance was very low; only five genera were observed with relative abundance greater than 0.1%, namely *Methanolinea* (0.8–1.32%), unclassified *Methanolegulaceae* (1.02–1.65%), *Methanothrix* (0.50–093%), *Methanobacterium* (0.10–0.71%) and *Methanomethylovorans* (0.09–0.17%) as listed in Table 2. Bacterial genera predominated, such as unclassified *Syntrophobacteriaceae* (6.88%) and the family *Syntrophobacteraceae* (7.10%) belonging to the *Proteobacteria* and *Synerigestetes* phyla, respectively, the genus *Aminivibrio* (7.22%) and family *Synergestaceae* (10.47%) belonging to the phylum *Synergistetes*. During the steady-state period, the TVFAs concentration was below 60 mg/L and the COD removal in the AnMBR reached above 91%, while the microbial community was roughly stable (Figure 3). At the genus level, on days 140, 162 and 190, *Methanolinea* (5.26–6.45%) belonging to hydrogenotrophic methanogens predominated, except on day 162, which showed a low population of *Methanolinea* of 1.58% (Table 2). Interestingly, the family Methanobacteriaceae exhibited low relative abundance during the steady-state period, but its population increased after shock-loading.

The significant level of hydrogenotrophic methanogens played an essential role in the conversion of VFAs during the steady-state period of the AnMBR. *Methanomatrix* (*Methanosaeta*) was present at a low level of 0.50–0.93% at start-up and its population clearly increased (1.36–3.07%) during the steady-state period (Table 2); however, *Methanosarcina* was insignificant at all operational stages. The genera unclassified *Methanolegulaceae* and *Methanospirillum* showed an increase (Table 2). At this steady-state stage, on days 140, 162 and 190, the predominant bacteria genera were unclassified *Betaproteobacteria* (13.07%, 37.65% and 15.18%), unclassified *Bacteria* (19.68%, 9.95%, and 16.73%), and unclassified *Rhodocyclaseae* (10.57%, 0.22%, and 9.56%), respectively.

During the steady-state period, VFAs removal was largely dependent on hydrogenotrophic methanogens (family *Methanoregulaceae* and genus *Methanolinea*, unclassified *Methanoregulaceae*), and the aceticlastic methanogens (family of *Methanotrichaceae* and genus of *Methanothrix*) (Table 2 and Figure S3). The hydrogenotrophic methanogens, genus *Methanolinea* (5.26–6.45%), dominated over aceticlastic methanogens, genus *Methanomatrix* (*Methanosaeta*) (1.36–3.07%) (Table 2); the acetate generated was probably oxidized by syntrophic acetate-oxidizing bacteria to generate hydrogen, which was used for methane generation Karakashev et al., [21]. On day 210, the methanogen population was completely changed, with the genera dropping below 0.9%; interestingly, *Methanobrevibacter* (0.55%) was the only methanogen observed with increasing relative abundance under these harsh conditions.

The abrupt shock loading provoked an increase of *Bacteroidetes* (*Bacteroidaceae*) and *Firmicutes* (*Clostridia*), respectively. The genus level composition was dominated by unclassified *Bacteria* (19.73%), *Bacteroides* (17.04%), unclassified *Bacteroidetes* (5.60%), *Clostridium sensu stricto* (2.00%), unclassified *Ruminococcaceae* (1.59%) and unclassified *Lachnospiraceae* (2.17%) belonging to the *Firmicutes* phylum, unclassified *Porphyromonadaceae* (4.42%), and unclassified-bacteroidales belonging to the *Bacteroidetes* phylum. At the family level, after unclassified family (45.30%), the family of *Bacteroidaceae* (17.04%) and *Porphyromonadaceae* (4.65%) belonging to phylum *Bacteroidetes*, and *Acidaminococcaceae* (3.00%), *Ruminococcaceae* (1.74%), *Clostridiaceae1* (2.45%) and *Lachnospiraceae* (2.21%) belonging to phylum *Firmicutes*, were represented in high relative abundance (Figure S2). At the class level, *Flavobacteria* (*Bacteroidetes*) and *Negativicutes* (*Firmicutes*) appeared (Figure 3). In this study, a new inoculum from the Xiao Hong Men municipal wastewater treatment plant was used to speed up the recovery process. The inoculum composition (Seed 2) was dominated by *Euryarchaeota* (family and genus *Methanobacteriaceae* and *Methanobacterium* respectively), *Bacteroidetes* and *Firmicutes*.

A methanogen population shift in the AnMBR was observed after day 210, where the relative abundance of methanogen increased dramatically as VFAs were consumed (Figure 2a). The aceticlastic and hydrogenotrophic methanogens were increased during the recovery stage (Table 2); however, the aceticlastic methanogens dominated over hydrogenotrophic methanogens, and *Methanomatrix* was the most abundant genus, which resulted in low acetate levels (acetate 20.04 ± 1.67 mg/L) compared to those in the steady-state period (acetate 22.60 ± 8.33 mg/L), when hydrogeno-trophic bacteria predominated (Table 2). The relative abundance and population of methanogens before overloading was partially recovered after overloading. The continuous mixing in the AnMBR used in this study, along with the high shear stress in the side-stream AnMBR membrane at cross flow velocity of 0.11 m/s, were suggested to affect the microbial community.

During the recovery period, the dominant families shifted from *Rhodocyclaceae*, *Chitinophagaceae*, *Pseudomonadaceae* to *Anaerolineaceae*, *Comamonadaceae*, *Enterobacteriaceae*, *Syntrophaceae* and the genera *Syntrophomonas* and *Clostridium* (Figure S2 and Table 2). At the steady-state period, genera *Desulfobrio* and *Sulphovum*, the families *Desulfovibrionaceae* and *Desulfomicrobiaceae* remained approximately below 0.5%. At this stage, the daily biogas accumulation rate was above 1.85 ± 0.45 L/day; however, during the shock loading stage, daily biogas production was completely suppressed. After a period of operation, the microbial community analysis indicated that the genera *Desulfobrio* (0.4–1.71%) and *Sulphovum* (0.17–0.39%) increased; however, the daily biogas accumulation remained below (0.48 ± 0.33 L/day). Besides, hydrogen sulfide gas was recorded at high concentration (Figure 2b), so that the shock-loading created environmental conditions beneficial for sulfate-reducing bacteria.

#### 3.2.3. Similarity, Richness and Diversity among AnMBR Microbial Community

**Figure 4:**
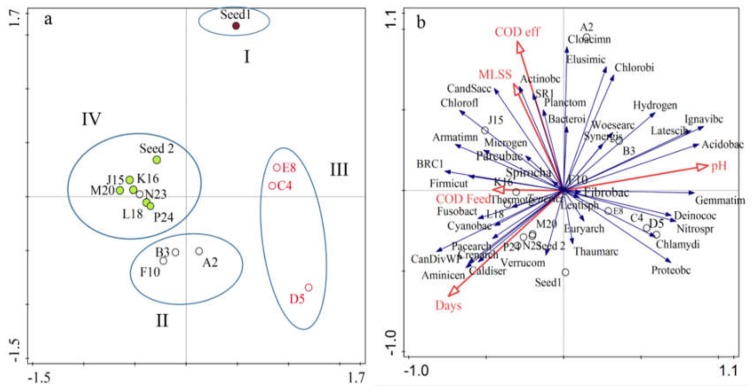
Principal component analysis (PCA) of microbial community at phylum level for samples (**a**); and Redundancy analysis (RDA) of microbial community and environmental factors (**b**).

As shown in Figure 4a AnMBR samples were clustered into four groups: (1) Group I contained one sample on day 0 (seed sludge); (2) Group II contained three samples on days 70 and 100 during the start-up period and day 210, during the overloading time, which presented similarity in terms of community; (3) Group III contained three samples on days 140, 162, and 190 during the steady-state period; (4) Group V presented six samples on days 230, 245, 250, 260, 275 and 285 during the recovery period (Figure 4a). Results showed that the samples on days 70, 100 and 210 were similar to each other and presented roughly similar populations of *Proteobacteria* and high relative abundance of *Bacteroidetes* and *Firmicutes* (Figure 3); similarity was also observed between the samples taken on days 140, 162 and 190, which have high relative abundance of *Proteobacteria* and *Euryarchaeota* in common, except on day 162. The samples collected during the recovery period on days 230, 245, 250, 260 and 285 shared the same populations of *Proteobacteria* and unclassified phyla.

These observations indicate that a distinct shift clearly occurred in the microbial populations of the AnMBR system. The clustering illustrated that operational conditions such as start-up, steady-state, overloading and recovery had a stronger selective effect on the microbial community composition than feed substrate, and showed the presence of dynamic community shifts. Dramatic shifts in the AnMBR microbial community were observed in both the archaeal and bacterial communities, and the abrupt overloading had a greater effect on the diverse population compared to the start-up period. The microbial communities in the AnMBR were analyzed to compare the bacterial richness, abundance of species and diversity. The alpha diversity index is shown in Table 3, including the Chao and Ace estimators, coverage, Shannon and Simpson diversity indexes. The Chao and Ace estimators, which emphasize community richness and abundance of species, were slightly low in the seed sludge (Seed1) from the brewery wastewater treatment plant. During the period of start-up, the Chao and Ace estimators increased to double the values of the seed sludge (Seed 1), from day 70, sample A2 to day 100, sample B3. During the steady-state stage, Chao and Ace values were mostly stable, as shown in Table 3, except for sample D5 on day 162, which presented low the OTU number of 866.62; the microbial community population changed slightly during this period, with an increase in *Bacteroidetes*, and the number of OTUs increased again in sample E8 to about 1360.16 on day 192. These results suggested an increase in the microbial richness and diversity, which enhanced the AnMBR performance.

After the period of AnMBR overloading, the sample F10 Chao and Ace estimators on day 210 increased to about 1536.00 and 1493.78, respectively. After re-inoculation the bioreactor with new seed sludge from Xiao Hong Men (Seed 2), which presented high Chao and Ace values (1319.10 and 1320.80), respectively, Chao and Ace increased sharply in sample J15 on day 230 to about 1652.04 and 1673.08, respectively. The values showed a slight decrease in sample K16 to about 1287.90 and 1354.73 on day 245 and sample L18 to about 995 and 1287.90 on day 250, respectively; however, Chao and Ace fluctuated in samples M20 on day 260 to about 1409.52 and 1438.39 and remained mostly constant in the next two samples N23 and P24 to about (1205.00 and 1221.05) and (1270.44 and 1313.02) respectively. In the recovery period, sample J15 on day 230 presented high richness and abundance of species compared with all other samples. The calculated Shannon diversity index revealed that the diversity of the microbial community was the highest in sample J15, followed by sample K16 (Table 3).

As shown in Figure S5, the rarefaction curves suggested that the sequencing depths for the 12 AnMBR samples and two seed samples were sufficient to cover the whole diversity. This was confirmed also by the coverage values of the two seed sludges and 12 AnMBR samples (Table 3). To distinguish the relationship between microbial communities and environmental variables, and examine the correlations among AnMBR samples, seed sludge microbial communities and explanatory variables, redundancy analysis (RDA) was used in Figure 4b. Seven variables including influent chemical oxygen demand (COD), effluent COD, AnMBR mixed liquor suspended solids (MLSS), exposure time and pH were considered (Table 1). At the phylum level, as indicated in Figure 4b samples J15, K16, L18 were linked to *Firmicutes* and *Chloroflexi*, however, these samples were negatively correlated to *Proteobacteria*. The pH and samples E8, C4, D5 were positively correlated to *Proteobacteria*, *Acidobacteria*, *Chlamydiae*, *Deinococcus*, *Nitrospirae*, *Gemmatimonadete* and *Ignavibacteriae*.

## 4. Discussion

### Microbial Community Shift and Contribution in the AnMBR

**Figure 5:**
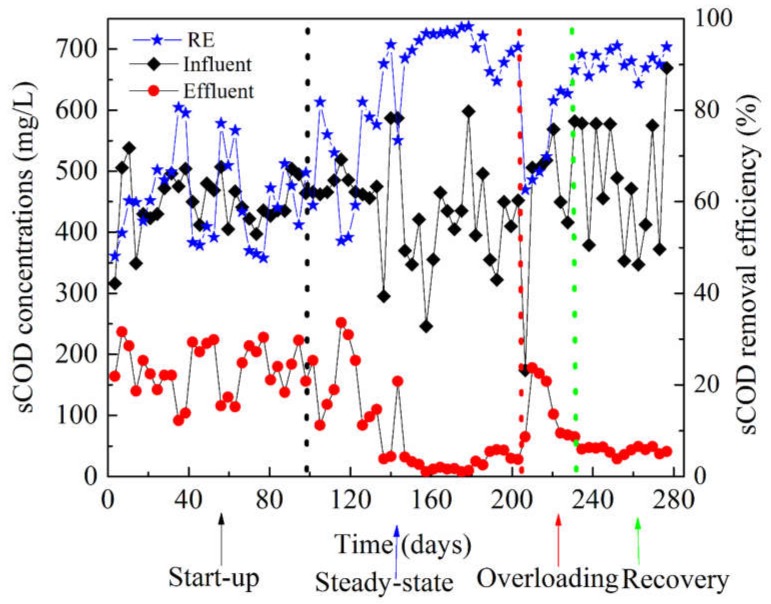
COD removals in the AnMBR process.

The relationship between microbial community shift and the COD removal performance in the AnMBR (Figure 5) was analyzed in this study. At the start-up operation, the COD removal increased from 50% to 70%, the ORP values decreased from −60 mV to −200 mV (Table 1). Similarly, total TVFAs concentration was decreased from 170 mg/L to 70 mg/L, towards the steady-state. During the start-up and overloading operation, the VFAs including acetic acid, propionate and butyric acids exhibited the high peak value in the AnMBR. The genus of microbial community that are reported to occur in start-up and unstable processes predominated, such as *Bacteroides* [22]. *Bacteroidetes* have been reported to be saccharolytic and sugar fermentative bacteria, which generate propionate and acetate in anaerobic bioreactors [23], and this suggested that the high acetate and propionate levels accumulated in the AnMBR during the start-up and overloading periods were probably produced by the high relative abundance of *Bacteroidetes* observed in this study. Previous research pointed out the key members of the microbial community that exhibit high relative abundance during shock loading such as Bacteroidetes [24], Firmicutes [22,25]. Studies reported different VFAs used as indicator for monitoring the anaerobic digestion process. For example, propionate was used as a key indicator of the anaerobic digestion process in steady-state [26], and a previous study illustrated that the propionate to acetate ratio can be used as an indicator for anaerobic digestion status, such as for start-up and steady-state, a suitable value for this ratio should be below 1.4 [27]. However, Pullammanappallil et al. revealed that a high propionate concentration was not sufficient to accurately monitor anaerobic digestion imbalance [28]. Li et al. illustrated that acetate exhibited a faster response to disturbance than propionate [10]. In addition, Kleyböcker et al reported that butyric acid and iso-butyric acid were suitable as control indicators of anaerobic digestion imbalance at the very early stage [29]. The propionate to acetate ratio fluctuated in the range of 0.2 to 1.58 in the start-up period and this value fluctuated in the range of 0.60 to 1.41 as the process achieved steady-state, propionate to acetate ratio at the critical value of 1.4 reported, did not show the imbalance in AnMBR operation and good performance in organic matter removal above 91% was achieved, Figure 5.

**Figure 6:**
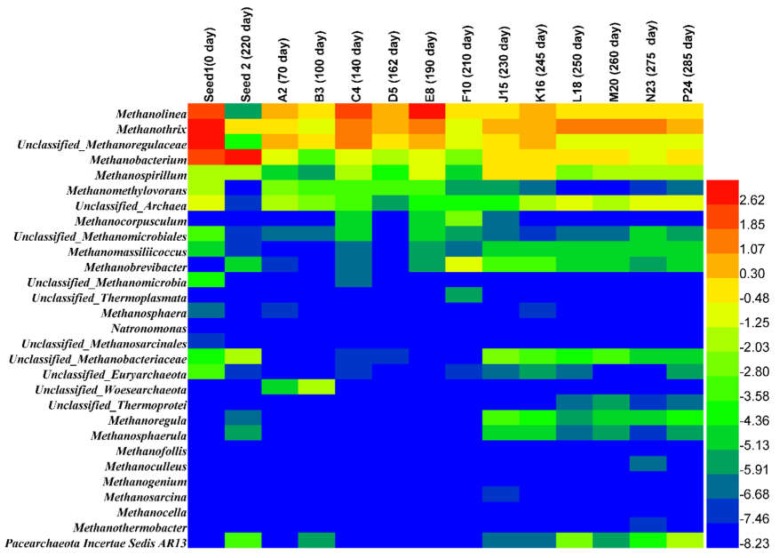
Heat map of *Archaeal* community compositions at genus level (the value values were log2 transformed).

On days 60 and 100 of the start-up period, the methanogens were predominated by the hydrogenotrophic genera of *Methanolinea* and Unclassified *Methanoleguraceae* (Figure 6), which were reported to be resistant to stress [30]. The VFAs in the AnMBR were suggested to be removed by syntrophic oxidation of acetate to carbon dioxide and hydrogen, and the hydrogenotrophic methanogen genera of *Methanolinea* and Unclassified *Methanoleguraceae* contributed to the hydrogen and carbon consumption [30]. Cho et al. (2013) have indicated that the predominance of hydrogenotrophic genera in the start-up was due to their greater tolerance to toxicity than aceticlastic methanogens [31]. At this stage, unclassified methanogen genera contributed a great deal to COD removal in the AnMBR (Figure 5). The family *Propionibacteriaceae* belonging to the phylum *Actinobacteria* (1.35–1.51%) exhibited high relative abundance and have been reported to generate propionic and acetic acid from glucose [32], which is suggested that they contributed to the acetic and propionic acids accumulation in the AnMBR during the start-up period.

However, at the steady-state and recovery periods, the acetic acid and butyric acid decreased gradually (Figure 2a), but the propionate persisted throughout the AnMBR operation; this was also reported in a previous study [7]. The ORP was −310 to −344 mV and −364 to −387 mV in the steady-state and recovery operation, respectively, and total VFAs concentration was below 70 mg/L. The phylum *Proteobacteria* predominated and showed to govern the AnMBR better performance of COD removal.

Previous research reported the dominance of proteobacteria in the anaerobic digestion at the steady-state, and *Proteobacteria* phylum correlated to the consumption of small substrates such as propionate, butyrate and carbohydrates [33]. TVFAs to alkalinity ratio was also used to monitor the AnMBR operation in this study, and it was 0.24 at the start-up and overloading operation, near the critical level of 0.3 reported for anaerobic digestion [34]. During the steady-state and recovery periods, the TVFAs to alkalinity ratio was 0.11, in range of reported value for stable anaerobic digestion operation. Therefore the TVFAs to alkalinity ratio was used as an indicator of monitoring imbalance in the AnMBR operation in this study. 

Previous studies revealed that a suitable inoculum is necessary to not only increase the degradation rate of pollutants and shorten the start-up period [35], but also to speed up the anaerobic digestion performance with high relative abundance of methanogen population for VFA consumption [36]. The seed sludge with a high relative abundance of *Proteobacteria* and *Methanolinea* and *Methanothrix* genera belonging to the phylum *Euryarchaeota* (Table 2) was used to speed up the start-up the AnMBR. From the seed sludge at the beginning (0 day) to the start-up operation, the microbial community exhibited a major community shift, *Proteobacteria*, *Bacteroidetes* and *Chloroflexi* predominated. The abundance of unclassified, *Acidobacteria*, *Bacteroidetes* and *Proteobacteria* in the start-up operation in this study were consistent with a previous study [37]. The occurrence of *Firmicutes* (genus of *Clostridium sensu stricto*) and *Actinobacteria* in the AnMBR at both start-up and overloading operation, showed their contribution to the hydrolysis and acidogenesis, similar to previous reported [38]. Figure 6 shows the archaea genera shift from the start-up, steady-state, overloading and recovery periods of the AnMBR. *Methanoregulaceae* was the dominant family during the steady-state period, and this family of hydrogenotrophic methanogens is reported to gain energy from the reduction of CO_2_ with H_2_ [39]. Syntrophic oxidation of acetate to carbon dioxide and hydrogen gas was suggested to take place, and hydrogenotrophic methanogens contributed to the conversion of hydrogen and carbon dioxide to methane [30]. Karakashev et al. reported that at insignificant levels of aceticlastic methanogen, acetate conversion is shifted to the hydrogenotrophic pathway [21], which is in agreement with this study. During the steady-state operation, VFAs removal was largely dependent on hydrogenotrophic methanogens, family *Methanoregulaceae* and genus *Methanolinea*, unclassified *Methanoregulaceae*, and aceticlastic methanogens, family *Methanotrichaceae* and genus *Methanothrix* (Figure 6).

The family *Methanotrichaceae* is reported to use only acetate as its substrate [40]. The hydrogenotrophic methanogens dominated over aceticlastic methanogens (Figure 6). In the AnMBR process, Aminovibrio genus was observed, Honda et al. (2013) reported Aminovibrio to be a genus that degrades amino acids and organic acids [41].

**Figure 7:**
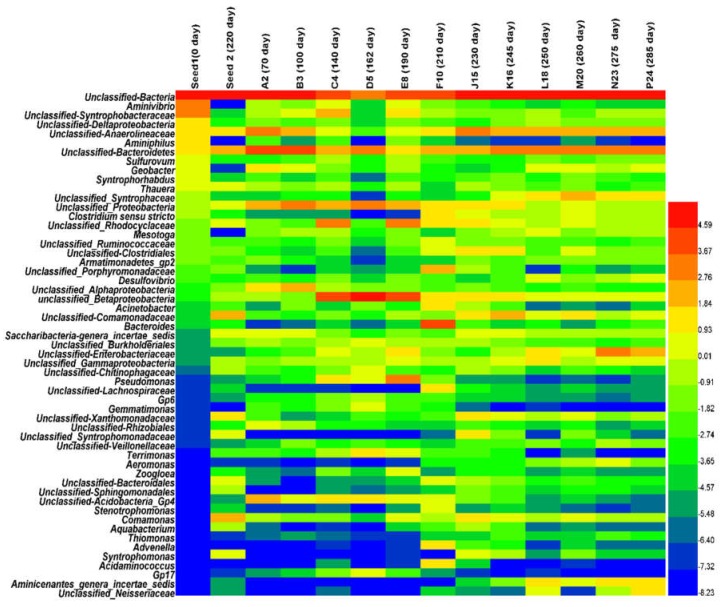
Heat map of bacterial classification at the genus level (the value values were log2 transformed).

When the AnMBR faced the hydraulic overloading due to the technical failure of the logic programe (Figure S1) on day 210, the hydraulic shocks resulted in a serious dropping of the COD removal efficiency from 91% to 60% (Figure 5). Decrease of *Proteobacteria* populations and increase of *Bacteroides* genus were observed as shown in Figure 7, the *Bacteroide* genus belonging to the phylum of *Bacteroidete* and *Clostridium sensu stricto* genus belonging to the phylum of *Firmicutes* were reported to be responsible of high accumulation of TVFAs in the AnMBR [42]. The pH was slightly stable and did not show clear response to the VFAs accumulations in the AnMBR (Table 1), the peak value of TVFAs concentration reached above 136 mg/L. However, the ORP showed response to the hydraulic shock loading with an increasing trend of ORP from −387 mV to −226 mV. The growth and activity rates of microorganisms such as methanogen community were inhibited with an increases of substrate at the shock hydraulic in the AnMBR process, and the biogas generation was suppressed (Figure 2c). It took two weeks for the AnMBR system to reach the recovery. *Acidogenic* bacteria exhibited rapid conversion of organic matter in the anaerobic digestion process, and are reported to have five-fold higher bacterial yield (gVSS/gCOD) compared to the methanogen community [43]; hence, the sudden rise in the OLR during the shock loading period resulted in the accumulation of VFAs. Acetate, propionate and butyrate have been reported to be the main VFAs produced in the overloading process [43].

During this study, syntrophic bacteria was reported to play a significant role during shock loading, according to Ketheesan and Stuckey syntrophic bacteria consume propionate and butyrate [44]. Interestingly, at the archaeal community, hydrogenotrophic methanogens, genus of *Methanobrevibacter* showed an increase in its population only at the shock loading period (Figure 6). Ziganshin et al. illustrated that *Firmicutes* are commonly known as fermenters and syntrophic bacteria that can consume volatile fatty acids such as propionate, acetate and butyrate in anaerobic digestion [45], thus a high relative abundance of *Firmicutes* was observed in the AnMBR during the shock loading period on day 210 and was correlated to the accumulation of acetate, butyrate and propionate. *Spirochetes* are reported to be bacteria that utilize hydro carbohydrates as their sole energy source for growth [46] that employ the syntrophic acetate oxidation process. The findings of this study were in agreement with previous researches to some extent, since this phylum appeared around day 210, when the acetate and carbohydrate concentrations were significantly high. The appearance of these species belonging to *Firmicutes* and *Bacteroidetes* at high VFAs concentrations has been previously reported [22]. A previous research study illustrated that after shock loading, it is advisable to add new inoculum rich in *Methanosarcina* to re-establish proper conditions for methanogen activity [47]; however, the inoculum used in the AnMBR from Xiao Hong Men was rich in hydrogenotrophic methanogens in this study.

During the recovery period, *Chloroflexi* and *Firmicutes* have been reported to be indicators of recovered systems [37]. However, in this study, the only stable community was *Chloroflexi*, and *Firmicutes* fluctuated during the recovery period (Figure 3). *Chloroflexi* are reported to be glucose-utilizing bacteria [48], which is in agreement with this study of the AnMBR, since the performance of COD removal was above 91% and acetate-utilizing methanogen populations were low in the AnMBR, so most of the pollutants were removed through the bacterial pathway. On day 230, the VFAs concentration in the AnMBR was high and the families *Anaerolineaceae* and *Comamonadaceae*, belonging to *Anaerolineae* and *Betaproteobacteria* respectively, became dominant, which have been reported to feed on propionate, acetate and butyrate [48]. The continuous mixing in the AnMBR in this study, along with the high shear stress in the side-stream tubular membrane at cross flow velocity of 0.11 m/s, has been reported to affect the methanogen community [49,50]. Methanogen communities are very sensitive to this type of mechanical force, as they need to be very close to hydrogen-producing bacteria in order to keep the hydrogen partial pressure low [49,50]. The observation of methanogen groups illustrated that both acetoclastic and hydrogenotrophic methanogen were present in the AnMBR. *Methanomatrix* (*Methanosaeta*), belonging to acetoclastic methanogens, represented the dominant pathway after overloading, while hydrogenotrophic bacteria dominated during the steady-state period (Figure 6). *Methanosaeta* was reported to be a dominant aceticlastic methanogen at low acetate concentrations [5,51], which is similar to the results of this research (Figure 2b). The methanogen community analysis indicated that the community did not recovery the previous structure and population, this shift of community affected the biogas production in the AnMBR (Figure 2c).

Sulfate-reducing bacteria were observed in the AnMBR process, and during the steady-state period, low relative abundance of sulfate-reducing bacteria was observed and the biogas accumulation rate was above 1.85 ± 0.45 L/day (Figure 2c). However, after the overloading period, the biogas accumulation rate declined and high hydrogen sulfide concentrations was recorded (Figure 2c). The genus of *Acinetobacter* presented high relative abundance at the overloading operation (Figure 7), in concurrence with high sulfur hydrogen detection in the AnMBR (Figure 2b). Previous research reported the *Acinetobacter* genus to oxidize the sulfur hydrogen (H_2_S) [38]. At the recovery stage, the peak value of biogas production reached a low level, but the COD removal reached again above 91%. These results suggested that methanogens and sulfate-reducing bacteria competed for substrates such as acetate, and sulfate-reducing bacteria have been reported to be more versatile than methanogens at consuming acetate [52]. The sulfate-reducing bacteria such as *Desulfobrio* and *Sulphovum* increased after the overloading period (Figure 6). This observation suggested another pathway of COD consumption, because sulfate-reducing bacteria are reported to compete with methanogens for common substrates [53]. The competition between methanogens and sulfate reducers for hydrogen and acetate substrates, are reported to result in biogas reduction [20]. The organic matter (chemical oxygen demand) could be assigned not only for methane production but also for the sulfate reduction pathway, with hydrogen sulfide as the end product [54]. The *Geobacter* genus exhibited high relative abundance above 1.9% at the start-up period and decreased below 0.01% at the steady-state and overloading operation, however, its population increased during the recovery period from 0.82% to 1.49%. *Geobacter* was reported to coexist with methanogens and play an essential role in electron transfer during the reduction of carbon dioxide to methane [55].

## 5. Conclusions

A lab scale anaerobic membrane bioreactor (AnMBR) with a side-stream tubular membrane was developed to treat synthetic domestic wastewater to evaluate the performance and characterize the microbial shifts of archaea and bacteria during the start-up, steady-state, overloading and recovery operation periods at mesophilic temperature (37.07 ± 1.287 °C). The AnMBR was successfully operated with an organic removal efficiency above 91% with effluent COD below 50 mg/L. During start-up, the organic matter removal was below 60%, the TVFAs to alkalinity ratio was established as an indicator of AnMBR imbalance, and a high abundance of *Bacteroidetes*, *Acidobacteria* and *Firmicutes* was observed. During the steady-state period, a significant shift occurred with *proteobacteria* as the most abundant phylum, and hydrogenotrophic methanogens dominated over aceticlastic methanogens. The propionate to acetate ratio was below 1.3 and the TVFAs to alkalinity ratio was below 0.08, and was correlated with good performance of organic matter removal above 91% in the AnMBR. In the shock loading period, the organic matter removal decreased from 91% to 60% and a significant shift was observed among bacterial and archaeal populations, with the dominance of *Bacteroidetes* and *Firmicutes*. *Methanobrevibacter* was the only observed archaea with population increased. The TVFAs to alkalinity ratio was 0.24, near the critical value of 0.3. In the recovery period, a significant shift occurred, especially in the methanogen group, which shifted to aceticlastic methanogens. Bacteria also shifted, however, neither bacteria nor archaea recovered their previous structure and population, which underlined the impact of shock loading on the community.

## Figures and Tables

**Table 1:** Operational and environmental variables.

Period	Samples	Sampling Period (d)	COD Feed (mg/L)	COD Effluent (mg/L)	MLSS (g/L)	ORP (mv)	pH	Alkalinity (mg/L CaCO_3_)
Startup	A2	70	455.00	173.47	6.73	−160.63 ± 55.75	7.72	529.00 ± 10.39
	B3	100	475.42	100.00	5.55	−174.45 ± 39.07	7.90	
Steady–state period	C4	140	380.50	17.00	5.45	−361.70 ± 30.62	7.97	675.25 ± 188.42
	D5	162	476	14.33	4.78	−310.88 ± 52.98	7.96	
	E8	190	391	34.66	5.09	−344.81 ± 26.42	7.77	
Overloading	F10	210	371.50	41.50	5.78	−226.28 ± 23.04	7.44	537.33 ± 238.28
	J15	230	500.80	153.0	5.89	−261.62 ± 37.55	7.40	
Recovery period	K16	245	504.12	76.50	6.22	−369.75 ± 29.90	7.29	758.00 ± 136.53
	L18	250	470.90	52.50	6.76	−364.00 ± 36.13	7.12	
	M20	260	509.58	42.66	5.33	−373.41 ± 30.20	7.36	
	N23	275	421.83	43.08	5.11	−387.69 ± 15.49	7.43	
	P24	285	582.33	43.83	4.77	−373.50 ± 14.00	7.26	

COD feed: chemical oxygen demand feed; COD effl.: chemical oxygen demand effluent; MLSS: mixed liquor suspended solids.

**Table 2:** The overall bacterial and archaeal community shift in the AnMBR.

Phyla and Genera Classification		Seed		Start-Up		Steady-State			Overloading		Recovery Operation				
Phyla	Genera	S1	S2	A2	B3	C4	D5	E8	F10	J15	K16	L18	M20	N23	P24
*Proteobacteria*	*Desulfovibrio*	0.22	0.08	0.23	0.13	0.46	0.05	0.41	0.21	0.06	0.23	1.03	0.42	0.71	1.21
	*Sulfurovum*	1.73	0.00	0.13	0.24	0.73	0.12	0.69	0.17	0.09	0.10	0.29	0.25	0.31	0.32
	*Comamonas*	0.00	4.58	0.79	0.29	0.44	0.05	1.01	0.87	2.53	1.88	0.93	1.24	0.65	0.87
	*Acinetobacter*	0.05	0.24	0.03	0.41	0.06	0.38	0.11	2.28	0.53	0.22	0.01	0.02	0.01	0.06
	*Geobacter*	1.62	0.00	1.93	1.56	0.52	0.09	0.53	0.12	0.06	0.10	1.49	1.05	0.82	1.36
	*Pseudomonas*	0.01	0.03	0.07	0.08	2.26	1.35	7.72	0.36	0.03	0.04	0.01	0.01	0.01	0.03
*Bacteroidete*	*Bacteroides*	0.04	0.11	0.00	0.01	0.05	0.01	0.07	17.04	0.15	0.06	0.11	0.03	0.13	0.24
*Firmicutes*	*Acidaminococcus*	0.00	0.00	0.00	0.00	0.02	0.00	0.01	2.00	0.05	0.00	0.00	0.01	0.00	0.00
	*Clostridium sensu stricto*	0.56	0.12	0.03	0.02	0.03	0.00	0.01	2.08	1.74	0.89	0.61	1.41	0.56	0.53
	*Syntrophomonas*	0.00	1.30	0.00	0.00	0.00	0.00	0.00	0.000	1.71	0.92	0.03	0.47	0.06	0.05
*Synergistete*	*Aminivibrio*	7.22	0.00	0.87	0.39	1.16	0.06	1.57	0.32	0.15	0.11	0.05	0.12	0.04	0.05
	*Aminiphilus*	2.37	0.00	0.17	0.02	0.25	0.00	0.33	0.04	0.01	0.00	0.00	0.02	0.00	0.00
*Acidobacteria*	*Gp17*	0.00	0.00	0.03	0.04	0.37	1.87	0.26	0.04	0.01	0.00	0.00	0.00	0.00	0.00
	*Gp6*	0.00	0.02	0.09	0.08	0.41	0.92	0.21	0.16	0.06	0.05	0.03	0.03	0.01	0.02
*Candidatus Saccharibacteria*	*Saccharibacteria*	0.03	1.28	1.62	1.07	0.49	0.08	0.39	0.22	1.02	1.15	0.70	0.85	0.86	0.70
*Euryarchaeota*	*Methanolin-ea*	4.68	0.02	1.32	0.87	5.24	1.58	6.45	0.92	0.94	1.28	0.77	0.88	0.85	0.76
	*Methanothrix*	6.29	0.92	0.93	0.50	3.07	1.36	2.12	0.46	2.05	1.87	2.34	2.33	2.35	1.71
	*Methanobac-terium*	5.51	7.06	0.71	0.10	0.59	0.28	0.47	0.23	1.21	1.16	0.92	0.86	0.64	0.80
	*Methanospi-rillum*	0.31	0.29	0.03	0.02	0.37	0.06	0.44	0.04	0.72	0.76	0.24	0.38	0.28	0.28
	*Methanomethylovorans*	0.28	0.00	0.18	0.09	0.10	0.11	0.08	0.02	0.02	0.01	0.00	0.00	0.00	0.01
	*Methanocorpusculum*	0.00	0.00	0.00	0.00	0.03	0.00	0.04	0.19	0.01	0.00	0.00	0.00	0.00	0.00
	*Methanobrevibacter*	0.00	0.03	0.00	0.00	0.00	0.00	0.01	0.55	0.12	0.12	0.02	0.04	0.01	0.03

**Table 3:** Diversity and Richness indices of microbial communities for the AnMBR samples.

No.	Days	Number of Sequences	OTU	Chao	Ace	Coverage	Shannon	Simpson
Seed1	0	29,443	609	743.10	745.92	0.994	4.199	0.034
A2	70	29,487	1108	1331.54	1341.14	0.990	5.054	0.019
B3	100	29,660	1017	1231.55	1257.01	0.990	4.628	0.035
C4	140	29,107	1073	1388.16	1392.34	0.988	4.790	0.025
D5	162	29,597	679	866.62	874.25	0.993	3.816	0.094
E8	190	29,535	1002	1360.16	1355.55	0.988	4.519	0.035
F10	210	29,230	1211	1536.00	1493.78	0.988	5.060	0.032
Seed2	220	29,244	1041	1319.10	1320.80	0.989	4.182	0.083
J15	230	29,372	1399	1802.48	1810.62	0.985	5.275	0.021
K16	245	29,452	1305	1652.04	1673.08	0.986	5.111	0.027
L18	250	29,622	995	1287.90	1354.73	0.989	4.953	0.015
M20	260	29,645	1088	1409.52	1438.39	0.988	4.841	0.029
N23	275	29,532	924	1205.00	1221.05	0.990	4.728	0.023
P24	285	29,553	993	1270.44	1313.02	0.989	4.917	0.017

* Chao: Community richness estimator. A higher number represents more richness; Ace: abundance of species; Shannon: Diversity index.; Good’s coverage: Sampling depth.

## Supplementary Materials

The following are available online at http://www.mdpi.com/1660-4601/15/7/1399/s1, Figure S1: AnMBR logic control program. Figure S2: The heat Heat map showing the relative abundances of various bacteria at family level (>0.01%) across all 12 AnMBR and two inoculum samples. Figure S3: The heat Heat map showing the relative abundances of various *Methanogen* at family level (>0.01%) across all 12 AnMBR and two inoculum samples. Figure S4: *Archaea* and *Bacteria* showing the relative abundances at class level (>1%) across all 12 AnMBR and two inoculum samples. Figure S5: Chao (a), Coverage (b), Rarefaction curve (c) and Shannon curve (d). Table S1: The relative abundances (%) of various *Bacteria* and *Archaea* at phylum level across all 12 AnMBR and two inoculum samples.
