# Supplementary material for: Characterization and Dynamic Shift of Microbial Communities during Start-Up, Overloading and Steady-State in an Anaerobic Membrane Bioreactor
Source: Int J Environ Res Public Health. 2018 Jul 3;15(7):1399. doi: 10.3390/ijerph15071399 (PMC6068774; doi:10.3390/ijerph15071399)
Supplement: Supplementary file 1 [file ijerph-15-01399-s001.pdf]

Appendix A. Supplementary data

Article

Characterization and dynamic shift of microbial communities during start-up, overloading and stead-state in anaerobic membrane bioreactor

Nsanzumukiza Martin Vincent [1,2,3], **Yuansong Wei** [1,2,3,4,*], **Junya Zhang** [1,2,3], **Dawei Yu** [1,2,3], **and Juan Tong** [1,2,3]

[1] State Key Joint Laboratory of Environmental Simulation and Pollution Control, Research Center for Eco-Environmental Sciences, Chinese Academy of Sciences, Beijing 100085, China; nsanzumumartiv_st@rcees.ac.cn (N.M.V.); yswei@rcees.ac.cn (Y. Wei); zjyzjjzmt@163.com (J.Z.); dwyu@rcees.ac.cn (D.Y.); hittj@163.com (J.T.)
[2] Department of Water Pollution Control Technology, Research Center for Eco-Environmental Sciences, Chinese Academy of Sciences, Beijing 100085, China
[3] University of Chinese Academy of Sciences, Beijing 100049, China
[4] Institute of Energy, Jiangxi Academy of Sciences, Nanchang 330096, China
* Correspondence: yswei@rcees.ac.cn; Tel.: +86-10-6284-9690

Received: xx May 2018; Accepted: XX June 2018; Published: date

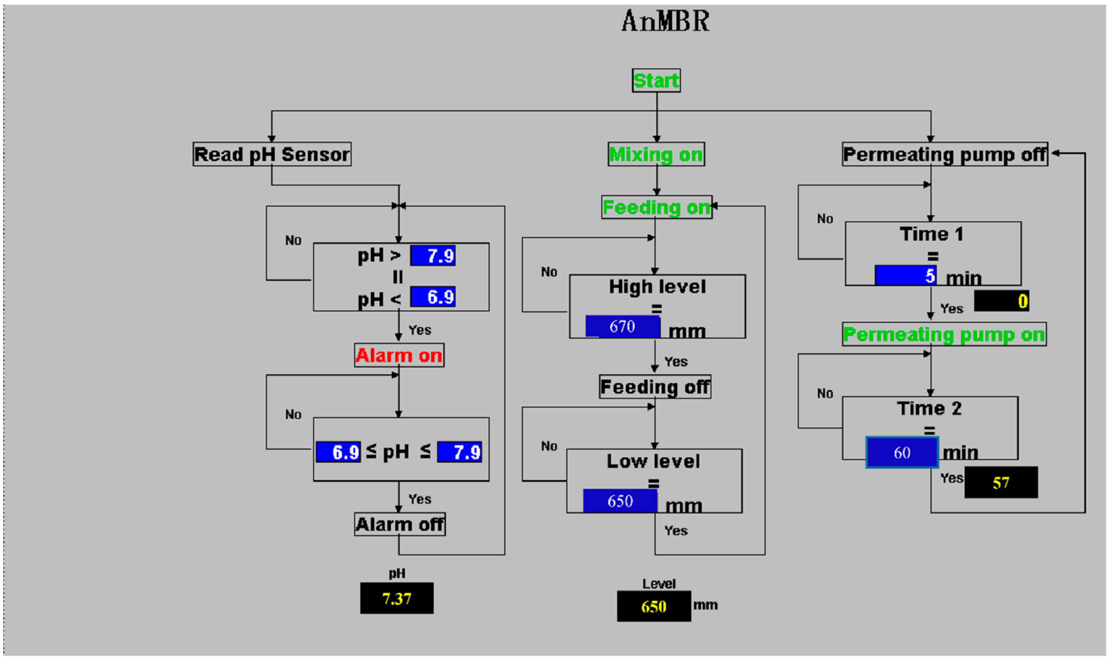

Figure S1. AnMBR logic control program

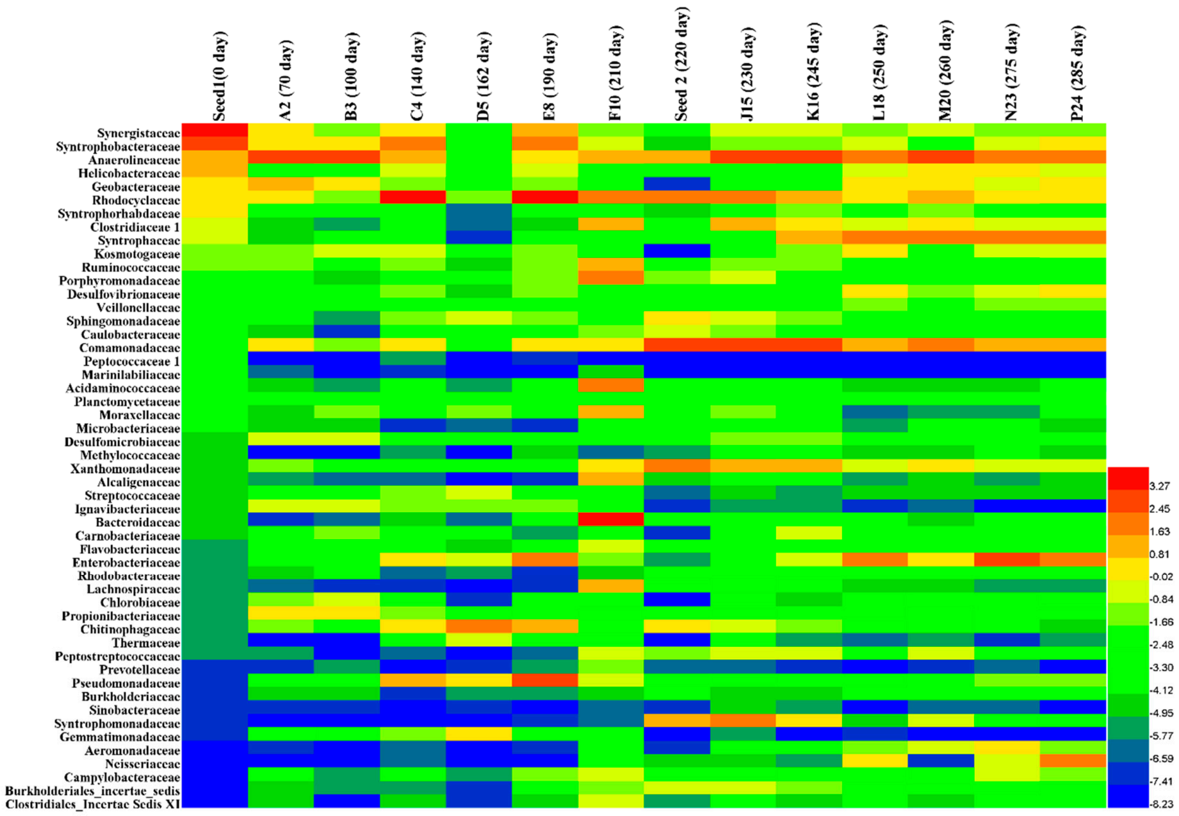

Figure S2. The heat Heat map showing the relative abundances of various bacteria at family level (>0.01%) across all 12 AnMBR and two inoculum samples.

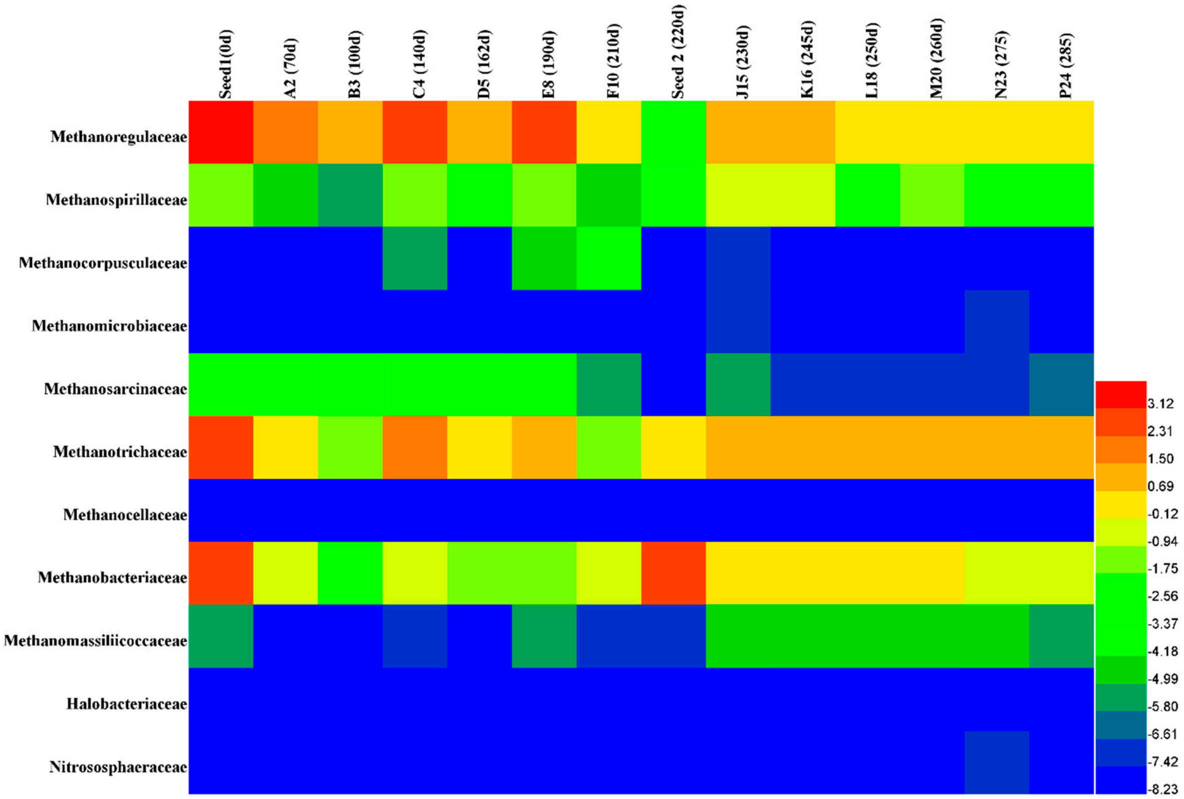

Figure S3. The heat Heat map showing the relative abundances of various Methanogen at family level (>0.01%) across all 12 AnMBR and two inoculum samples.

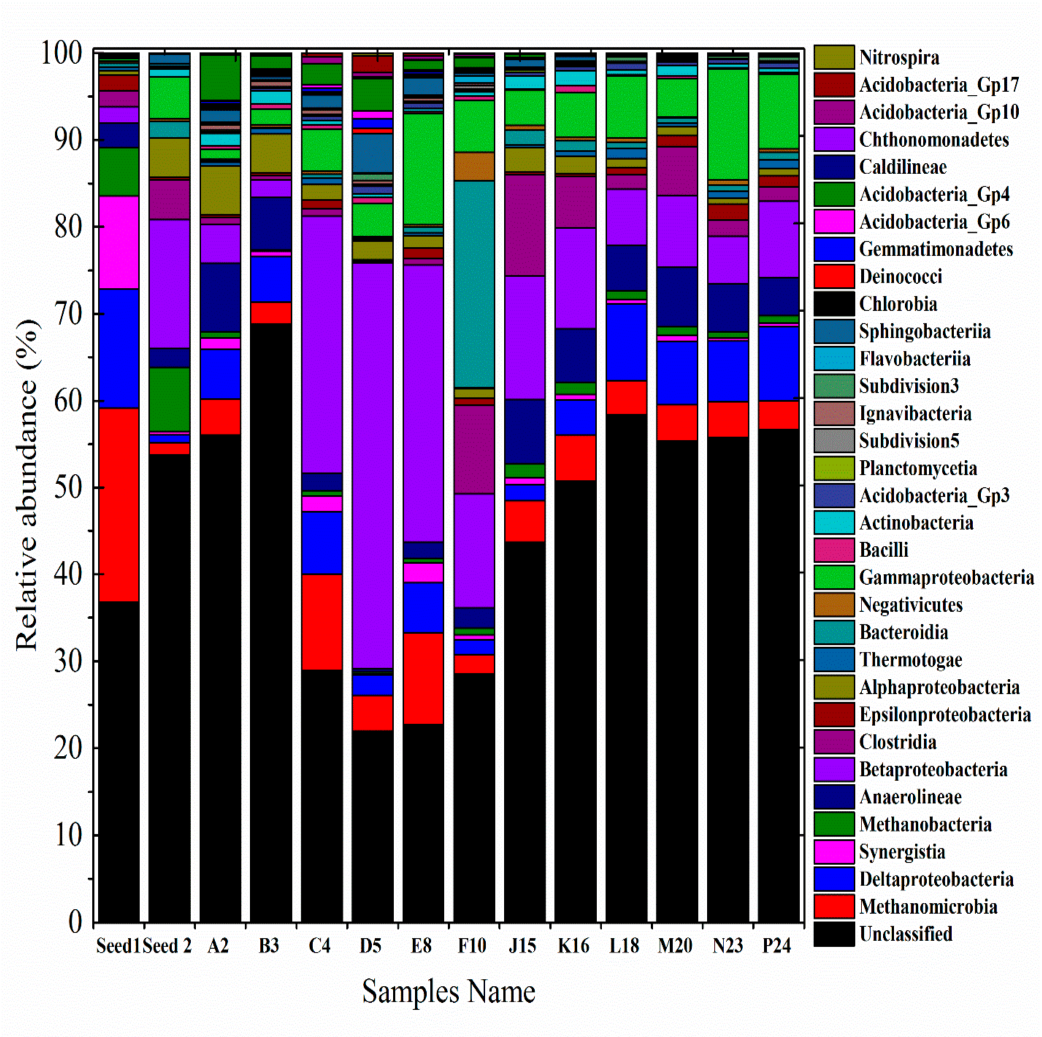

Figure S4. Archaea and Bacteria showing the relative abundances at class level (>1%) across all 12 AnMBR and two inoculum samples.

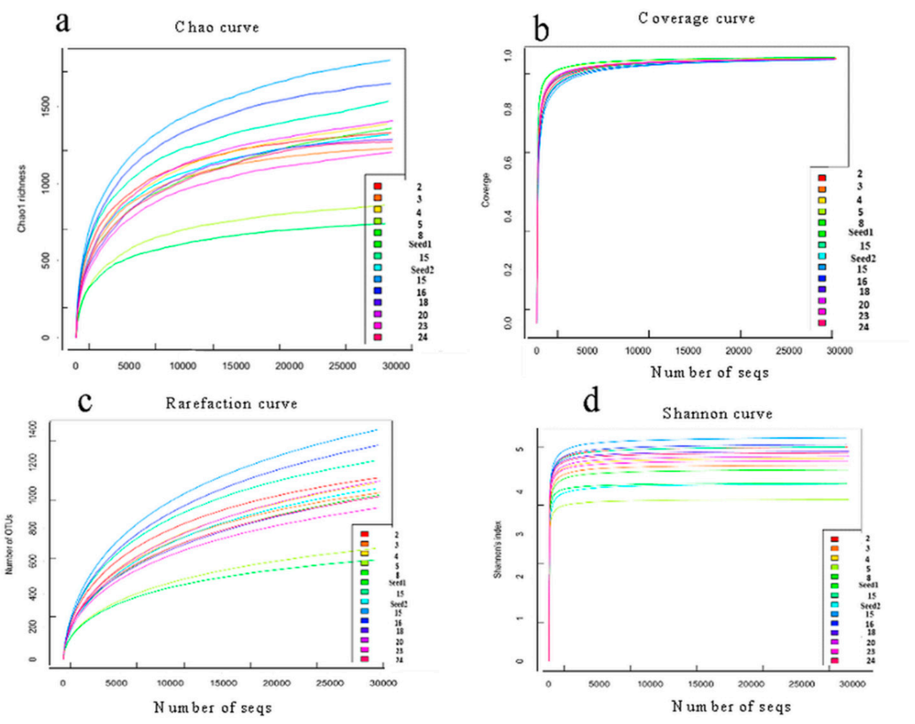

Figure S5. (**a**) Chao; (**b**) Coverage; (**c**) Rarefaction curve and (**d**) Shannon curve.

Table S1. The relative abundances (%) of various bacteria and archaea at phylum level across all 12 AnMBR and two inoculum samples.

Phyla	S1	S1	A2	B3	C4	D5	E8	F10	J15	K16	L18	M20	N23	P24
Proteobacteria	18.85	26.74	22.13	25.78	46.53	57.42	54.35	23.86	25.07	25.10	24.91	23.36	28.43	28.55
Euryarchaeota	28.08	8.81	4.86	2.65	11.67	4.44	11.10	3.09	6.42	6.77	5.03	5.21	4.85	4.24
Bacteroidetes	2.53	7.31	21.07	19.21	6.55	13.16	5.74	30.05	8.03	11.08	12.76	11.86	10.54	12.10
Acidobacteria	0.15	0.15	5.77	2.21	4.60	8.07	2.70	2.03	0.92	0.79	0.97	0.59	0.61	0.84
Synergistetes	10.67	0.32	1.32	0.58	1.73	0.08	2.29	0.55	0.84	0.61	0.50	0.68	0.37	0.41
Chloroflexi	2.89	2.18	8.48	6.70	2.28	0.54	2.05	2.53	7.71	6.35	5.48	7.18	5.75	4.62
Firmicutes	2.46	5.65	1.92	1.76	1.85	1.11	1.39	16.05	13.30	7.71	2.56	6.38	2.83	2.52
Ignavibacteriae	0.06	0.01	0.58	0.64	0.55	0.46	0.44	0.23	0.03	0.02	0.01	0.01	0.00	0.00
Candidatus Saccharibacteria	0.03	1.31	1.64	1.08	0.50	0.09	0.41	0.22	1.03	1.17	0.71	0.86	0.87	0.71
Thermotogae	0.44	0.02	0.50	0.68	0.74	0.11	0.34	0.07	0.35	0.60	1.22	0.34	0.79	1.05
Actinobacteria	0.10	0.94	1.45	1.57	0.48	0.34	0.33	0.45	1.52	1.71	0.49	1.22	0.45	0.46
Gemmatimonadetes	0.01	0.00	0.30	0.14	0.42	1.07	0.30	0.10	0.03	0.00	0.01	0.00	0.00	0.00
Verrucomicrobia	0.11	0.13	0.18	0.16	0.23	1.00	0.22	0.54	0.43	0.43	0.70	0.29	0.32	0.63
Chlorobi	0.03	0.00	0.55	0.61	0.13	0.01	0.20	0.21	0.06	0.04	0.16	0.06	0.08	0.10
Deinococcus-Thermus	0.02	0.01	0.10	0.03	0.18	0.61	0.14	0.14	0.07	0.03	0.02	0.02	0.01	0.03
Planctomycetes	0.06	0.21	0.25	0.16	0.18	0.23	0.14	0.14	0.27	0.20	0.19	0.22	0.13	0.18
Armatimonadetes	0.35	0.57	0.56	0.18	0.12	0.15	0.11	0.06	0.60	0.34	0.46	0.65	0.45	0.36
Nitrospirae	0.00	0.00	0.02	0.06	0.06	0.34	0.06	0.03	0.00	0.01	0.00	0.00	0.00	0.00
Chlamydiae	0.00	0.03	0.01	0.00	0.06	0.15	0.04	0.02	0.04	0.03	0.00	0.02	0.01	0.00
Spirochaetes	0.02	0.06	0.02	0.01	0.01	0.01	0.04	0.75	0.13	0.03	0.01	0.02	0.01	0.00
Hydrogenedentes	0.15	0.00	0.05	0.03	0.05	0.00	0.03	0.01	0.01	0.00	0.00	0.02	0.01	0.00
Latescibacteria	0.00	0.00	0.05	0.02	0.03	0.05	0.02	0.02	0.00	0.00	0.00	0.00	0.00	0.00
Fibrobacteres	0.00	0.00	0.00	0.00	0.00	0.00	0.01	0.00	0.00	0.00	0.00	0.00	0.00	0.00
Cloacimonetes	0.02	0.03	0.02	0.01	0.01	0.00	0.01	0.00	0.01	0.01	0.00	0.00	0.00	0.00
Lentisphaerae	0.00	0.00	0.00	0.00	0.01	0.00	0.00	0.04	0.00	0.00	0.00	0.00	0.00	0.00
Cyanobacteri	0.00	0.01	0.00	0.00	0.00	0.00	0.00	0.00	0.00	0.00	0.00	0.00	0.00	0.01
Tenericutes	0.00	0.00	0.00	0.00	0.00	0.00	0.00	0.09	0.00	0.00	0.00	0.00	0.00	0.00
SR1	0.01	0.01	0.15	0.00	0.00	0.00	0.00	0.01	0.00	0.05	0.03	0.03	0.05	0.04
BRC1	0.01	0.02	0.00	0.00	0.00	0.00	0.00	0.00	0.03	0.02	0.02	0.01	0.00	0.01
candidate division WPS-1	0.00	0.00	0.00	0.00	0.00	0.00	0.00	0.01	0.01	0.01	0.01	0.01	0.01	0.01
Elusimicrobia	0.03	0.00	0.03	0.01	0.01	0.00	0.00	0.00	0.00	0.00	0.01	0.00	0.00	0.00
Fusobacteria	0.00	0.04	0.00	0.00	0.00	0.00	0.00	0.00	0.00	0.00	0.00	0.00	0.00	0.01
Aminicenantes	0.00	0.04	0.00	0.00	0.00	0.00	0.00	0.00	0.07	0.37	2.23	1.42	2.41	1.97
Microgenomates	0.01	0.03	0.06	0.01	0.00	0.00	0.00	0.00	0.02	0.04	0.03	0.06	0.04	0.03
Caldiserica	0.00	0.00	0.00	0.00	0.00	0.00	0.00	0.00	0.00	0.02	0.08	0.05	0.06	0.09
Parcubacteria	0.00	0.00	0.07	0.00	0.00	0.00	0.00	0.00	0.00	0.00	0.06	0.00	0.00	0.10
Pacearchaeota	0.00	0.09	0.00	0.02	0.00	0.00	0.00	0.00	0.01	0.01	0.22	0.02	0.07	0.29
Woesearchaeota	0.00	0.00	0.04	0.39	0.00	0.00	0.00	0.00	0.00	0.00	0.00	0.00	0.00	0.00
Crenarchaeota	0.00	0.00	0.00	0.00	0.00	0.00	0.00	0.00	0.00	0.00	0.01	0.02	0.01	0.01
Uniclassified	32.92	45.26	27.81	35.27	21.01	10.57	17.53	18.68	32.98	36.45	41.12	39.36	40.84	40.61
